# Targeting IL-6 Trans-Signaling: Amplifying the Benefits of IL-6 Inhibition in Myocardial Infarction∗

**DOI:** 10.1016/j.jacbts.2021.01.007

**Published:** 2021-05-24

**Authors:** Pål Aukrust, Ola Kleveland, Lars Gullestad

**Affiliations:** a Faculty of Medicine, Institute of Clinical Medicine, University of Oslo, Oslo, Norway; b Section of Clinical Immunology and Infectious Diseases, Oslo University Hospital Rikshospitalet, Oslo, Norway; c Research Institute of Internal Medicine, Oslo University Hospital Rikshospitalet, Oslo, Norway; d Clinic of Cardiology, St. Olavs Hospital, Trondheim University Hospital, Trondheim, Norway; e Department of Cardiology, Oslo University Hospital Rikshospitalet, Oslo, Norway; f K.G. Jebsen Cardiac Research Centre and Centre for Heart Failure Research, University of Oslo, Oslo, Norway

**Keywords:** infarct size, inflammation, interleukin-6, myocardial infarction, myocardial remodeling

**Figure:**
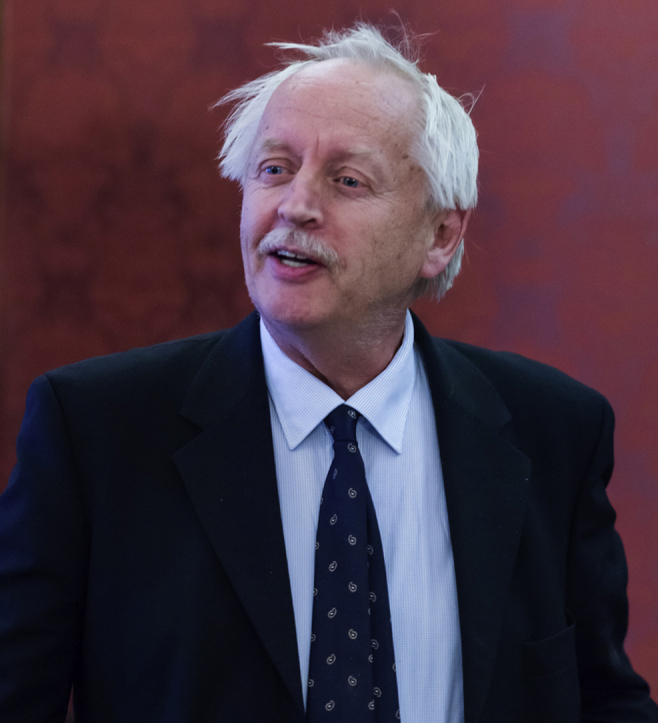

It is well established that atherosclerosis is a prototypical chronic inflammatory disorder, and there are several studies suggesting that persistent inflammation could also play an important pathogenic role in coronary artery disease and the development of chronic heart failure. This has encouraged several clinical studies targeting inflammation in these disorders. Most focus has been directed at treatment options that attenuate interleukin (IL)-1 and IL-6 signaling. Thus, in the large CANTOS (Canakinumab Antiinflammatory Thrombosis Outcomes Study), the authors showed that in patients with previous myocardial infarction (MI) and residual inflammatory risk (C-reactive protein >2 mg/l), treatment with canakinumab, a monoclonal antibody directed against IL-1β, led to a significantly lower rate of recurrent cardiovascular events as compared with placebo (1). Importantly, this benefit was achieved without affecting other risk factors, including lipid levels. Moreover, we have recently showed that IL-6 inhibition with tocilizumab, a humanized anti-IL-6 receptor monoclonal antibody that inhibits the binding of IL-6 to both membrane-bound and soluble IL-6 receptors, significantly attenuated inflammation and reduced troponin T release in patients with acute non–ST-segment elevation MI, in particular in those that underwent percutaneous coronary intervention (2). Furthermore, in a subsequent study among patients with acute ST-segment elevation MI, acute administration of tocilizumab before reperfusion with percutaneous coronary intervention increased myocardial salvage as measured by magnetic resonance imaging 3 to 7 days after the intervention (3).

However, modulation of cytokine signaling is not straightforward. First, these inflammatory cytokines are not per se “bad.” For example, IL-6 plays a significant role in myocardial remodeling predisposing to the development of overt heart failure. However, whereas experimental mice models have shown that enhanced IL-6 activation promotes myocardial hypertrophy, deficiency of its receptor induces severe cardiac dilation, illustrating the balance between beneficial and harmful effects of this cytokine, where too much and too little both can be harmful. Also, most inflammatory cytokines have some anti-inflammatory properties. Thus, tumor necrosis factor (TNF) is regarded as a prototypical inflammatory cytokine, but is also a potent inducer of the prototypical anti-inflammatory cytokine IL-10. Thus, experimental studies have shown that a too modest inflammatory response in the acute phase may predispose to a state of persistent inflammation by inducing a too weak counteracting anti-inflammatory response. Furthermore, intervention targeting cytokine signaling may have different effects depending on the molecular target of the actual medication. Thus, whereas much focus has been directed toward canakinumab that targets IL-1β, anakinra, an IL-1 receptor antagonist, blocks the interaction between both IL-1β and IL-1α with IL-1 receptor type 1 (IL-1R1). Notably, the role of IL-1α in atherogenesis has recently received some attention. Moreover, whereas etanercept, an Fc tagged form of the soluble TNF receptor type 2, binds to TNF and prolongs its half-life, infliximab, a monoclonal antibody against TNF, directly inhibits TNF, and when binding to membrane-bound TNF it can induce antibody-dependent cytotoxicity. These differences could influence their clinical effects and could have been of importance for the different effects of etanercept and infliximab in the pioneering studies on TNF inhibition in chronic heart failure (4).

In this issue of *JACC: Basic to Translational Science*, George et al. (5) further illustrate the complexity of cytokine intervention in cardiac disease. IL-6 has 2 distinct signaling pathways termed classic and trans. Whereas classic signaling is achieved by IL-6 binding to its membrane-bound α-receptor (mIL-6R), which associates with a dimer of the receptor β-subunit glycoprotein 130 (gp130) to initiate intracellular signaling, trans-signaling is initiated by the binding of IL-6 to the soluble form of the receptor (sIL-6R), and then this IL-6/sIL-6R complex subsequently binds to a dimer of membrane-bound gp130. It seems that trans-signaling to a lesser degree than classical signaling influences the anti-inflammatory properties of IL-6. Moreover, because gp130 in contrast to mIL-6R is ubiquitously expressed, this pathway enables IL-6 to stimulate all tissues*.* In the present study, the authors hypothesized that the novel recombinant fusion-protein sgp130Fc would be more efficacious than pan-antagonism through anti-IL-6 antibodies, because of its more specific targeting of the selective proinflammatory trans-signaling pathway (5). Indeed, in a model of reperfused MI in male rats they show that sgp130Fc reduced infarct size and preserved cardiac function 28-days post-MI as compared with anti-IL-6 therapy. They suggest that this difference was caused by selective blockade of trans-signaling and thereby more selective anti-inflammatory effects by the fusion-protein sgp130Fc. There are some previous studies on the effects of blocking trans-signaling as compared with blocking total or classical IL-6 signaling. It has been showed that selective inhibition of IL-6 trans-signaling, but not by classical IL-6 inhibition, significantly improved the fracture healing outcome after combined injury. Moreover, it was recently shown that IL-6 classic signaling via the membrane-bound IL-6R is responsible for the defense of the body against *Listeria* and mycobacteria infection, and that this defense is not affected by the selective blockade of IL-6 trans-signaling further illustrating the differences between these 2 IL-6 signaling pathways.

In the present study the authors extend these previous findings by showing beneficial effects of blocking trans-signaling, as compared with pan-antagonism (blocking trans and classical signaling simultaneously), on the development of MI size and post-MI remodeling in an experimental rat model. Their data suggest that these findings were related to decreased infiltration of neutrophils and in particular monocytes into the myocardium and enhanced apoptosis within the myocardium. These data are intriguing and novel and illustrate that detailed knowledge of cytokine signaling and network is prerequisite in the field of cytokine modulation therapy in cardiovascular disease.

Their study also has some limitations and challenges for future research. As the authors state, the experiments were performed in a rat model and the kinetic of IL-6 following MI in these animals seems to be different from that seen in humans (biphasic vs. persistent elevation). Moreover, the anti-inflammatory effects of blocking trans-signaling in their post-MI model are not well characterized. The phenotypes of the infiltrating macrophages will be crucial (resolving vs. inflammatory) in particular because resolving macrophages is of major importance to remove apoptotic cells (efferocytosis). Moreover, they show enhanced apoptosis of noncardiomyocyte cells, but it is important to learn whether these cells reflect fibroblasts, endothelial cells, or infiltrating leukocytes, or a combination thereof. Finally, the wide distribution of trans-signaling enabling IL-6 to stimulate all tissues could be beneficial, but it could also result in more side effects in tissues not involved in the targeted process. Nonetheless, it is noteworthy that in several substudies of our tocilizumab non–ST-segment elevation MI study (2), we failed to demonstrate any strong anti-inflammatory effects of tocilizumab and we proposed that the beneficial effects of tocilizumab in this population were not related to secondary effects on inflammation, but rather direct effects on IL-6 signaling on for example matrix remodeling.

In relation to anti-inflammatory intervention in MI, including those targeting IL-6 signaling, there is a need for larger studies with clinical endpoints. The work by George et al. (5) also underscores the need for more mechanistic studies including studies on attacking different targets in the cytokine signaling pathway as illustrated by modulation of IL-6 signaling. To get several steps ahead it is also of major importance to explore why an intervention worked or did not work by performing in-depth studies in parallel to clinical studies.

## Funding Support and Author Disclosures

All authors have reported that they have no relationships relevant to the contents of this paper to disclose.
